# Controlled Plasmonic Coupling in Silver Nanoplate Dimers for Enhanced Plasmonic Sensing

**DOI:** 10.3390/nano16080486

**Published:** 2026-04-19

**Authors:** Lucrezia Catanzaro, Marcello Condorelli, Mario Pulvirenti, Luisa D’urso, Giuseppe Compagnini

**Affiliations:** 1 Department of Chemical Sciences, University of Catania, 95125 Catania, Italy; lucrezia.catanzaro@phd.unict.it (L.C.); luisa.durso@unict.it (L.D.); gcompagnini@unict.it (G.C.); 2 Istituto Nazionale Scienza e Tecnologia dei Materiali (INSTM), 95124 Catania, Italy

**Keywords:** plasmonics, nanophotonics, self-assembly, optical sensing

## Abstract

Noble metal nanostructures provide versatile platforms for light manipulation through localized surface plasmon resonances (LSPRs). Among them, triangular silver nanoplates (AgNPTs) exhibit strong field-enhancement and spectral tunability, yet assembling them reproducibly on solids is challenging. We report a two-step functionalization strategy for constructing ordered AgNPT dimers on silica substrates, combining 3-aminopropyltriethoxysilane (APTES) anchoring with 1,4-butanedithiol bridging. AFM reveals face-to-face dimers with well-defined sub-nanometer gaps. Large-area AFM statistics collected over multiple regions (N = 80 nanoplates per condition) confirm reproducible and selective vertical dimerization. Extinction spectroscopy reveals sequential dielectric and coupling effects: thiol adsorption red-shifts the main resonance from 700 to 780 nm because of increased local refractive index and near-field damping, whereas dimerization partially restores it to ≈750 nm, consistent with plasmon hybridization within rigid ∼0.7 nm molecular gaps, where nonclassical moderation may occur but classical hybridization fully explains the observed shifts. Concomitantly, the extinction intensity doubles, following an exponential growth toward saturation during assembly. Surface-enhanced Raman scattering (SERS) measurements using 4-mercaptobenzoic acid (4-MBA) confirm a fourfold increase in the SERS enhancement factor from monolayer to bilayer, consistent with near-field coupling and hotspot formation at interplate junctions. Quantitative plasmon sensitivity analysis yields comparable results between experiments and finite-difference-time-domain simulations, confirming that the observed spectral shifts arise from near-field coupling and dielectric modulation rather than ensemble effects. This reproducible methodology enables precise tuning of NPT orientation, spacing, and optical response, providing a robust platform for enhanced sensing, SERS, and nanophotonic device engineering.

## 1. Introduction

In recent decades, the investigation of localized surface plasmon resonances (LSPRs) in noble metal nanostructures has fundamentally transformed the fields of nanophotonics and molecular sensing [1]. Among the various morphologies explored, triangular silver nanoplates (AgNPTs) have emerged as particularly compelling candidates due to their exceptional spectral tunability, which can be precisely shifted from the visible to the near-infrared (NIR) region by modulating their aspect ratio [2]. The anisotropic geometry of AgNPTs, characterized by sharp vertices, provides natural “antenna” effects that lead to significant electromagnetic field enhancement, often surpassing the performance of spherical nanoparticles in refractometric sensing and surface-enhanced Raman scattering (SERS) applications [3].

However, the optical properties of isolated nanostructures are frequently eclipsed by the performance of coupled plasmonic systems. When two AgNPTs are positioned within nanometric proximity, the interaction of their evanescent near-fields induces a profound reconfiguration of the LSPR profile, a phenomenon often described through the plasmon hybridization model [4]. This coupling not only leads to the formation of electromagnetic “hotspots” within the interparticle junctions, which enable single-molecule detection [5,6] levels, but also causes a significant red shift and spectral broadening of the resonance peaks [7,8]. These spectral transformations are highly sensitive to the interparticle distance and orientation, governed by the “plasmonic ruler” equation [9,10,11].

Despite this potential for extreme sensitivity, the reproducible and controlled fabrication of ordered NPT dimers on solid substrates remains a significant challenge. Conventional self-assembly methods often lack the necessary precision to control the interparticle gap, resulting in heterogeneous optical responses and unpredictable spectral broadening that compromise the reliability and signal-to-noise ratio of nanophotonic devices [12].

In this context, achieving a uniform assembly is essential to overcome ensemble averaging effects and harness the full potential of coupled near-fields. Some traditional methods for assembling NPs include: (1) DNA origami, which enables the fabrication of complex assemblies, but is expensive and presents several challenges in the design of specific DNA sequences, as well as potential assembly errors [13,14]. (2) Polymer templating, in which NPs are embedded within a polymer matrix. Even though the method is simple, it reduces NPs’ sensitivity to the surrounding environment, thereby limiting their application in optical sensing [15]. (3) Electron beam lithography, which represents an alternative strategy that allows precise control over NPs’ assemblies, as long as the feature sizes remain above the technique’s resolution limits (a few nanometers) [16]. By contrast, the APTES/dithiol strategy adopted in this work provides a simple, robust, and scalable bottom-up route for the direct assembly of AgNPT dimers on solid substrates, enabling rigid sub-nanometer gap definition, controlled face-to-face stacking, and straightforward integration with large area optical characterization. Unlike more elaborate templating or lithographic approaches, this method combines experimentally accessible surface chemistry with statistically verifiable structural uniformity, thereby offering a particularly effective platform for reproducible plasmonic sensing, SERS, and substrate-supported nanophotonic applications.

In this work, we report a robust two-step functionalization strategy for the bottom-up construction of ordered AgNPT dimers on silica substrates. Our approach combines the use of 3-aminopropyltriethoxysilane (APTES) as a primary anchoring layer with 1,4-butanedithiol as a molecular bridge to induce face-to-face dimerization. Using atomic force microscopy (AFM) and extinction spectroscopy, we characterize the sequential effects of changes in the dielectric environment and plasmonic coupling. Despite the technique having been widely recognized for nearly two decades, to our knowledge, face-to-face dimer assemblies of anisotropic silver NPTs directly on a solid substrate through thiol linkers have not been previously reported, and no prior work has reported their optical response, plasmonic sensitivity, or SERS performance under substrate-supported, rigid-gap conditions.

Specifically, we demonstrate that while thiol adsorption induces a pronounced red shift due to local refractive index increase, subsequent dimerization partially restores the resonance position and doubles the extinction intensity. This behavior is consistent with plasmon hybridization across rigid ∼0.7 nm dithiol linkers, where the bonding dipole mode shifts to higher energy and the response reflects strong near-field coupling rather than uncontrolled aggregation, allowing for the precise tuning of the optical response [17,18,19]. The efficacy of this platform is further validated through SERS measurements and finite-difference time-domain (FDTD) simulations, providing a comprehensive framework for the design of high-performance plasmonic platforms.

## 2. Materials and Methods

### 2.1. Synthesis of AgNPTs

AgNPTs were synthesized via a seed-mediated growth procedure following previously reported protocols [20,21]. This method allows precise control over nanoparticle size, thickness, and shape, which are critical parameters for tuning their plasmonic response. The initial seeds were generated by chemical reduction of silver precursors in aqueous solution under controlled temperature and stirring conditions. Growth of triangular nanoplates was promoted by adding shape-directing agents, which favor anisotropic growth along specific crystallographic facets [22].

The purified AgNPTs were stored until further use, and their size and shape distributions were characterized by transmission electron microscopy (TEM) before substrate immobilization.

### 2.2. Substrate Preparation and Surface Functionalization

Glass substrates were thoroughly cleaned via sequential sonication in acetone, ethanol, and deionized water, followed by an inverse piranha treatment to remove organic contaminants and generate hydroxyl-terminated surfaces.

The substrates were subsequently functionalized with APTES in aqueous solution. Silanization introduces amine groups onto the substrate surface, providing positively charged anchoring sites for the negatively charged AgNPTs [23]. The functionalization parameters, including silane concentration and reaction time, were optimized to achieve homogeneous monolayers.

**Figure 1:**
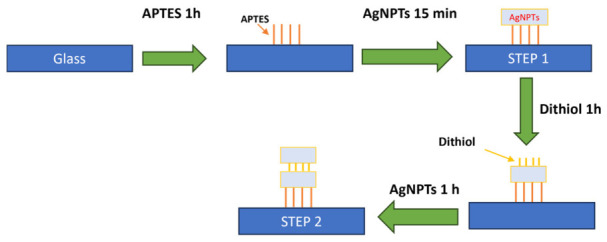
Sketch of the main steps involved in the fabrication of NPT dimers on a silica substrate.

Immediately after silanization, the APTES-coated substrates were immersed in the AgNPT colloidal suspension for 15 min (Figure 1, Step 1). The extent of nanoparticle deposition was controlled by adjusting immersion time and nanoplate concentration, enabling systematic tuning of surface coverage and preventing uncontrolled aggregation. To ensure statistical robustness, AFM measurements were performed over multiple, spatially separated areas, and height distributions were extracted from N = 80 nanoplates per configuration (monomer and dimer).

### 2.3. Controlled Dimer Formation via Thiol Linkers

To achieve controlled dimer formation, the immobilized AgNPTs were further functionalized with bifunctional 1,4-butanedithiol linkers. Similar alkanedithiol-mediated plasmonic assemblies are well documented for noble metal nanoparticles [24]. Before the dithiol functionalization step, the substrates were treated with 0.1 M NaOH for 1 h as a cleaning step. The samples were subsequently rinsed with deionized water and dried under nitrogen before the dithiol treatment. After cleaning, each sample was immersed in a 1 mM ethanolic solution of 1,4-butanedithiol for 1 h, allowing one thiol group to covalently bind to the immobilized NPT’s silver surface while leaving the second thiol exposed for subsequent NPT attachment.

After thorough rinsing to remove physisorbed molecules, the substrates were re-immersed in the AgNPTs colloidal suspension for 1 h (Figure 1, Step 2). The exposed thiol groups provided selective binding sites for free AgNPTs in solution, facilitating stable, face-to-face dimer formation with well-defined interparticle gaps. For triangular nanoplates lying flat on the substrate, vertical stacking is the only possible dimerization pathway; thus, AFM height provides the direct structural signature distinguishing monomers (~12 nm) from dimers (~25 nm)

### 2.4. Characterization

The morphology and spatial arrangement of the resulting nanostructures were analyzed by using a Witec Alpha 300 RS atomic force microscope (AFM) operating in alternate contact mode with a force modulation tip (AC), with reflex coating, and a 2.8 N/m force constant, and 75 kHz resonance frequency.

Optical characterization of AgNPTs in both colloidal and solid states was carried out using a commercial Agilent Cary 60 UV–Vis spectrophotometer(Agilent, Via P. Gobetti 2/C 20063 Cernusco sul Naviglio Milano, Italy) in standard transmission configuration.

Plasmonic sensitivity was quantified in the liquid phase by measuring resonance wavelength shifts upon immersion in liquid media of various refractive indices, following well-established procedures [20,21].

Raman spectroscopy was used to assess the adsorption of 1,4-butanedithiol on immobilized AgNPTs, following established SERS protocols for thiolated molecules on silver nanostructures [25,26]. For all the measurements, an excitation wavelength of 532 nm was used; the spectra were acquired with 10 s of acquisition time for 10 accumulations, using a 100× objective of a Raman microscope (WITECH alpha 300 Wissenschaftliche Instrumente und Technologie GmbH, Ulm, Germany).

Subsequent SERS measurements of 4-MBA were conducted under controlled laser fluence to prevent photothermal damage, according to best practices in plasmon-enhanced vibrational spectroscopy [25,27]. To evaluate the SERS performance, the analytical enhancement factor (AEF) was calculated following the approach described by Le Ru and co-workers [28], according to Equation (1):

(1) $$EF=\frac{I_{SERS}∕C_{SERS}}{I_{NR}∕C_{NR}}$$

where *I_SERS_* and *I_NR_* are the Raman intensities measured under SERS and normal Raman conditions, respectively, and *C_SERS_* and *C_NR_* are the corresponding concentrations of the probe molecule. In the present work, the enhancement factor was determined using the 1590 cm^−1^ vibrational band of 4-MBA, measured both in the normal Raman spectrum and in the SERS spectrum under identical instrumental conditions. The intensity of this band was used as the reference signal for the EF calculation.

### 2.5. FDTD Simulations

FDTD simulations (2022 R2.4 FDTD software package) were carried out using Lumerical Ansys, following standard methodologies for modeling plasmonic nanoparticles [29].

Material parameters for silver were taken from the Johnson and Christy dataset [30].

Boundary conditions and mesh refinement were optimized according to established computational plasmonics guidelines [31], enabling accurate simulation of the extinction spectra and electric field enhancement of both single nanoplates (monomers) and dimers. The geometry of the simulated nanostructures was defined from the dimensions obtained by AFM, including both thickness and lateral size. In the dimer model, the interparticle separation was fixed at 0.7 nm, in agreement with the expected length of the 1,4-butanedithiol molecular spacer employed in the experimental assembly. Following convergence analysis, a mesh size of 0.2 nm was selected for the FDTD simulations.

## 3. Results

### 3.1. Morphology and Dimer Formation

**Figure 2:**
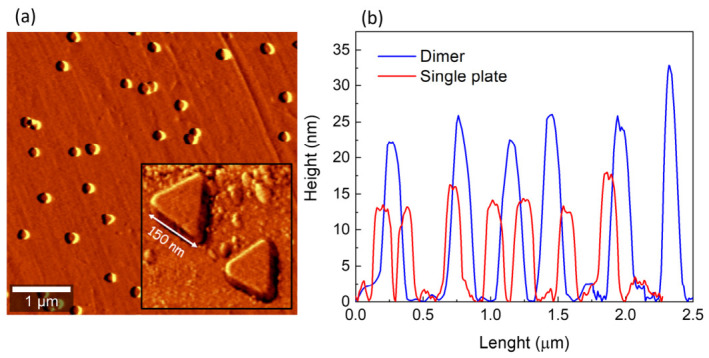
(**a**) AFM images of AgNPTs deposited on a silanized silica substrate after the initial immobilization step (monomers) and (**b**) AFM cross-section profiles before (red line) and after (blue line) dimer formation.

Figure 2 illustrates the stepwise assembly of AgNPT dimers on the functionalized silica substrate. Panel (a) shows an AFM image of the substrate after the initial immobilization step. The nanoparticles are randomly distributed across the surface but maintain uniform lateral dimensions. A higher-magnification inset highlights two individual NPTs, which lie flat on the substrate, confirming that the triangular basal plane is oriented face-down. This orientation is crucial, as it provides a well-defined surface for subsequent attachment of additional NPTs via thiol linkers.

Panel (b) presents AFM cross-section profiles taken along representative particles before and after dimer formation. In the initial deposition step, the NPTs exhibit an average height of ~12 nm, consistent with the thickness of single triangular nanoplates synthesized via the seed-mediated approach. Following exposure to the 1,4-butanedithiol linker and deposition of a second nanoplate, the cross sections show a systematic increase in height to ~25 nm. Figure S3 reports the statistical study of the size distribution of 80 nanoparticles, randomly analyzed over an area of 10 µm × 10 µm, for both substrates: monolayer (Figure S3a) and dimer (Figure S3b). The narrow height distribution centered at 25 ± 2 nm, observed for ~82% of the measured objects, demonstrates that the dimerization process is reproducible and selectively produces vertically stacked nanoplates over large substrate regions. This approximately two-fold height increase confirms the formation of stacked nanoplate dimers, with the second NPT anchored in a face-to-face “sandwich” configuration on top of the first. The uniformity of the height increase across multiple sampling regions indicates that dimerization proceeds in a controlled manner rather than via uncontrolled aggregation. These results provide direct structural evidence that the two-step functionalization strategy produces reproducible AgNPT dimers with well-defined orientation and interparticle spacing. Importantly, the random lateral distribution of particles does not contradict structural control: in this geometry, only the vertical (height) coordinate changes upon dimerization, while the lateral footprint remains unchanged.

### 3.2. Optical Properties and Plasmon Coupling

**Figure 3:**
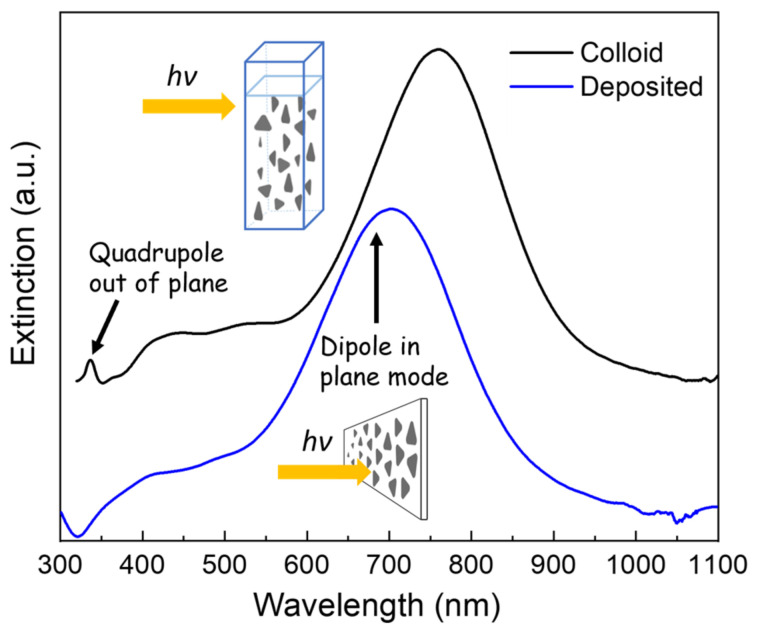
Representative extinction spectra of AgNPTs in colloid (black line) and deposited on silanized silica substrate (blue line).

The optical response of triangular AgNPTs was first evaluated in their colloidal state and compared to the spectra recorded after deposition on APTES-modified glass substrates (Figure 3). In aqueous suspension, the NPTs display a strong in-plane dipole plasmon resonance centered at around 800 nm, in agreement with previous reports for particles of comparable edge length and thickness [32]. Upon immobilization on the aminosilane-coated glass slides, the resonance undergoes a pronounced blue shift to ~700 nm. This spectral shift can be attributed to the change in the dielectric environment surrounding the nanoparticles: in the colloidal state, the NPTs are surrounded by water (n ≈ 1.33), whereas after deposition, they are in contact with air (n ≈ 1.0) and glass (n ≈ 1.5). The effective average refractive index sensed by the plasmon, therefore, decreases, leading to a net reduction in the resonance wavelength. Such environment-induced tuning of LSPR is well documented and highlights the strong sensitivity of AgNPTs to changes in the local dielectric medium [16].

An additional observation concerns the higher-order plasmon modes. In solution, the spectrum reveals a weak but distinct out-of-plane quadrupole resonance at ~330–340 nm. After deposition, however, this feature disappears almost entirely. This effect can be explained by the face-down orientation of the NPTs on the substrate surface, which restricts the excitation of out-of-plane oscillations. Because the quadrupole mode requires vertical displacement of conduction electrons relative to the basal plane, the intimate contact with the substrate effectively damps this resonance. Similar suppression of higher-order out-of-plane plasmon modes has been reported for flat-lying triangular nanoprisms immobilized on solid supports [33]. The persistence of only the dominant in-plane dipole resonance after deposition thus provides further confirmation of the NPT orientation inferred from AFM measurements.

To schematically illustrate the experimental conditions, the figure includes a cartoon depiction of the two configurations: a cuvette containing randomly dispersed NPTs in aqueous colloid, and a glass slide on which the NPTs are immobilized in a face-down orientation through the APTES functionalization layer. Together, these complementary structural and optical results establish a consistent picture of NPT geometry and plasmonic behavior before and after substrate deposition.

**Figure 4:**
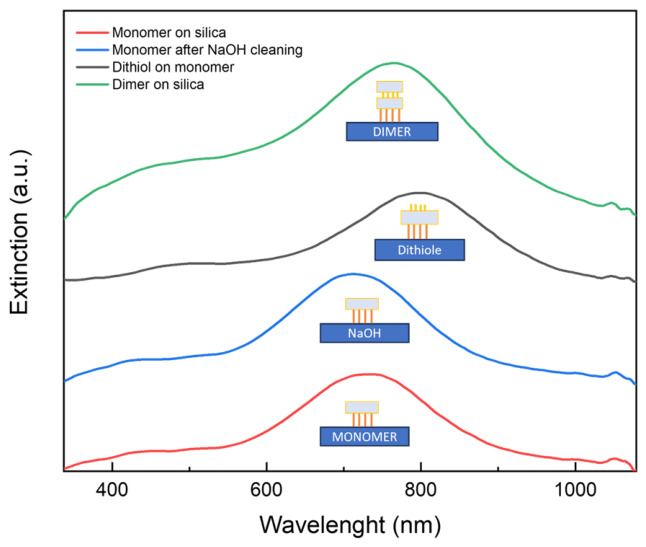
Extinction spectra of AgNPTs on a silanized silica substrate, measured immediately after deposition (red), after sodium hydroxide treatment (blue), following dithiol functionalization (black), and after dimer formation (green). Dashed lines are used as visual guides to highlight the shift in the plasmon peak.

The plasmonic response of the immobilized AgNPTs was monitored at each step of the dimer assembly process (Figure 4). The initial blue shift observed upon transferring AgNPTs from aqueous colloid (~800 nm) to the APTES-coated substrate ~720 nm originates exclusively from a change in the effective refractive index: immobilized nanoplates experience a lower average dielectric constant (air/glass) compared to water, leading to a reduced LSPR wavelength.

A brief NaOH treatment induces a minor additional blueshift (~15 nm), also attributable to a further decrease in the local refractive index due to the cleaning step.

Upon adsorption of the 1,4-butanedithiol linker, the resonance undergoes a pronounced redshift to ~780 nm. This spectral change arises solely from the formation of an organic molecular layer around the nanoplate, which replaces the surrounding air with an organic layer of higher refractive index (n ≈ 1.5–1.6) [34], thereby increasing the local dielectric constant and introducing moderate near-field damping [35]. No coupling effects are expected at this stage since each nanoplate remains isolated. Raman spectroscopy measurements (see Supporting Information, Figure S1) were performed after the 1,4-butanedithiol functionalization step and before the deposition of the second nanoplate. The spectra display characteristic C–S stretching and –CH_2_– vibration bands, confirming the successful adsorption of the dithiol molecules onto the silver surface [36]. The absence of additional features related to silver oxide or silane species further supports the selective attachment of the linker. These results provide molecular-level evidence for the formation of a uniform thiol layer, which serves as the bridging interface for subsequent dimer assembly.

**Figure 5:**
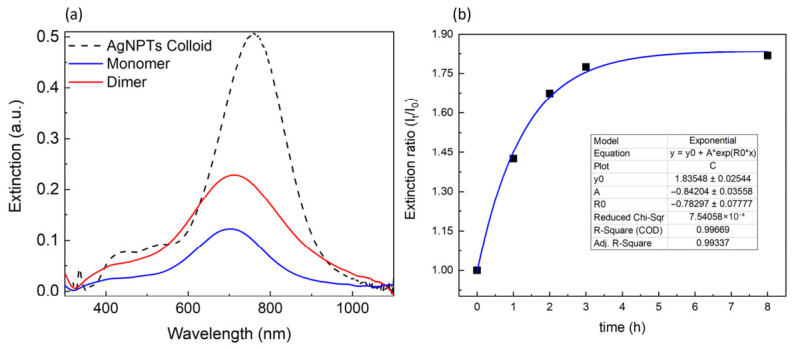
(**a**) Comparison of the extinction spectra of AgNPTs in liquid and on glass, as monomers and dimers. (**b**) Time evolution of the extinction intensity, expressed as the ratio of the measured extinction intensity to the maximum extinction intensity of the monomer, as a function of immersion time.

When a second nanoplate attaches in a face-to-face configuration through the dithiol linker, the plasmonic fields of the two plates hybridize within the rigid ~0.7 nm gap. This interaction partially counteracts the redshift previously induced by the dithiol, producing a blueshift from ~780 nm to ~740 nm. This shift is consistent with the formation of hybridized plasmon modes, where the bonding (in-phase) dipole mode shifts to higher energy due to coupling across the molecular spacer [19]. At this gap size, quantum tunneling effects may begin to moderate the interaction, but do not dominate the optical response, and classical hybridization remains sufficient to account for the observed spectral behavior. In addition to spectral position, the extinction intensity provides complementary information on the structural evolution of the system (Figure 5a). When comparing monolayer and bilayer assemblies of AgNPTs, the overall extinction magnitude at the dipolar resonance nearly doubles, while the spectral profile and linewidth remain almost unchanged. Importantly, the apparent absence of a spectral shift should be understood with respect to the resonance position of the initial substrate-supported monolayer: after the redshift induced by dithiol adsorption, dimer formation restores the resonance close to that original position. In this sense, the bilayer does not exhibit additional peak displacement or linewidth broadening indicative of uncontrolled aggregation or of a broad distribution of coupling strengths. This nearly linear scaling of intensity with the number of plasmonic elements, therefore, indicates that the addition of the second NPT layer proceeds in an ordered manner, rather than through random aggregation. The preserved line shape further suggests that vertical face-to-face stacking does not introduce significant inhomogeneous broadening of the in-plane dipolar plasmon mode, consistent with a controlled and homogeneous coupling regime rather than a disordered ensemble of interactions [31,32].

Notably, the recovery of the resonance toward the monolayer position also argues against effective geometric merging of the NPTs, which would otherwise alter the platelet aspect ratio and produce stronger spectral perturbations. Similarly, uncontrolled aggregation would be expected to generate broader and less reproducible spectral features. The observed behavior is instead consistent with well-defined dimers separated by a rigid, non-conductive molecular spacer, for which coupling occurs in a controlled fashion without aggregation-induced broadening.

Here, the presence of a uniform, non-conductive molecular subnanometer spacer preserves the optical individuality of each NPT, such that the bilayer response corresponds to the superposition of two weakly and homogeneously coupled plasmonic elements.

The kinetics of dimer formation were monitored by tracking the extinction intensity at the resonance maximum as a function of immersion time during the second NPT deposition (Figure 5b).

The temporal evolution follows a single-exponential growth, well described by the expression (2):

(2) $${I\left(t\right)=I}_{\infty }-∆Ie^{-kt}$$

where *I_∞_* represents the saturation intensity and *k* the apparent rate constant. This behavior is characteristic of Langmuir-type adsorption kinetics [37], in which the dimerization rate is proportional to the fraction of available binding sites on the linker-functionalized surface. The exponential approach to a plateau reflects progressive site occupation and steric saturation rather than uncontrolled aggregation. Accordingly, the process can be interpreted as a reaction-limited adsorption regime, where the formation of stable face-to-face dimers occurs through selective thiol-mediated binding between pre-anchored and free AgNPTs in solution.

### 3.3. Plasmonic Sensitivity

**Figure 6:**
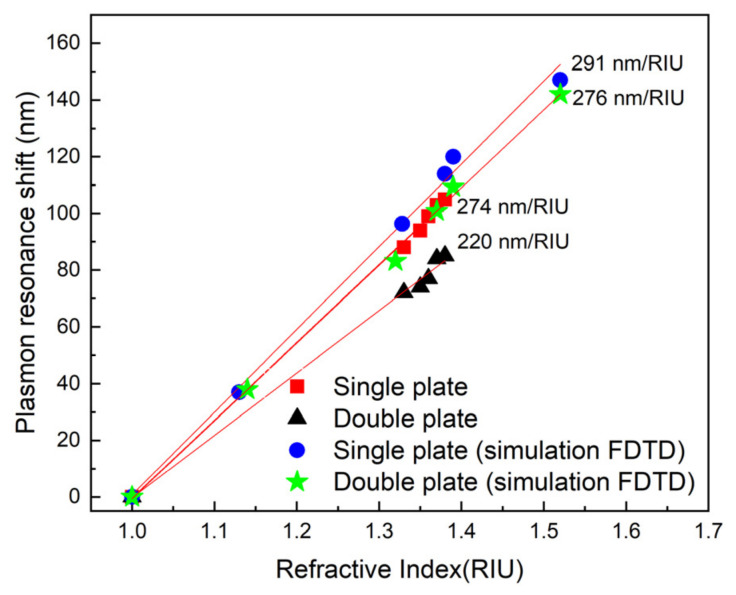
Comparison of refractive index sensitivity measured for monomers and dimers, alongside the sensitivity obtained from simulations of a single AgNPT.

The refractive-index dependence of the dipolar plasmon resonance was investigated to assess the sensitivity of the assembled AgNPT structures (Figure 6). For monolayer assemblies, a linear redshift of the dipolar peak was observed with increasing refractive index, corresponding to a plasmon sensitivity of 274 ± 5 nm/RIU. Upon formation of the bilayer (dimer) configuration, the sensitivity decreased slightly to 220 ± 7 nm/RIU, while the extinction magnitude nearly doubled, as previously shown in Figure 5. This result indicates that the dimerization process largely preserves the intrinsic refractive-index sensitivity of individual NPTs while providing a substantially stronger optical signal, which is advantageous for sensing applications limited by optical noise. Furthermore, the doubled extinction intensity improves the signal-to-noise ratio without spectral broadening, preserving the sensing figure of merit despite the modest reduction in wavelength sensitivity.

The comparison between experiment and simulation shows good agreement for the monomer, whose refractive-index sensitivity is 274 ± 5 nm/RIU experimentally and 291 ± 11 nm/RIU in the FDTD model (Figure S3a), corresponding to a difference in only about 6%. For the dimer, the simulated sensitivity is 276 ± 7 nm/RIU (Figure S3b), still close to the monomer response, whereas the experimental value decreases to 220 ± 7 nm/RIU. This indicates that the simplified numerical model reproduces the monomer behavior well but does not fully capture the sensitivity decrease observed experimentally after dimer formation. The discrepancy can reasonably be attributed to the idealized geometry assumed in the simulations, including perfectly defined nanoplate shape, uniform gap spacing, and a homogeneous dielectric environment, whereas the real system is affected by finite size dispersion, local substrate heterogeneity, slight deviations from perfect face-to-face alignment, and possible variations in linker packing and gap filling.

From the experimental point of view, the reduced dimer sensitivity can be rationalized by the stronger confinement of the plasmonic near field within the sub-nanometer interplate junction. In this configuration, a larger fraction of the electromagnetic energy is concentrated inside the dithiol-filled gap, where the local field is enhanced but the dielectric environment is less affected by changes in the external liquid medium. As a result, the effective sensing volume accessible to refractive-index variations is reduced, even though the local field intensity is increased. This interpretation is consistent with previous studies on coupled plasmonic dimers, in which reduced bonding-mode sensitivity has been linked to limited gap accessibility and to the fraction of the plasmonic field overlapping the perturbed dielectric region [38]. In our system, this stronger gap confinement enhances extinction and SERS while slightly reducing the wavelength shift per refractive-index unit. Importantly, the full width at half maximum (FWHM) of the experimental spectra remains essentially unchanged between monomer and dimer assemblies, indicating minimal broadening. This observation suggests that the dimers are well-defined and non-coalesced, with nearly perfect face-to-face alignment producing a near-ideal superposition of the plasmonic modes, as already mentioned. As a result, the figure of merit (FOM = sensitivity/FWHM) remains effectively the same for monomer and dimer structures, combining high sensitivity with enhanced extinction intensity. Such behavior is highly desirable for sensing applications, as it improves signal strength without sacrificing spectral resolution. This balance between preserved sensitivity, unchanged linewidth, and amplified optical signal is consistent with previous reports on coupled nanoparticle systems [39,40].

### 3.4. SERS Effect

To further evaluate the local field enhancement associated with the AgNPT assemblies, SERS measurements were performed using 4-MBA as a molecular probe. SERS spectra of 4-MBA exhibit several characteristic vibrational bands, including the intense aromatic C–C stretching mode at ~1580 cm^−1^ [28]. The band at 1076 cm^−1^ is generally adopted as the reference for quantitative analysis of the enhancement factor, as it arises from a combination of C–S stretching and in-plane ring vibrations and is comparatively insensitive to molecular orientation, surface charge, and protonation of the carboxylate group. In contrast, the 1580 cm^−1^ band may shift or vary in relative intensity depending on adsorption geometry and charge-transfer interactions with the silver surface. Therefore, the 1076 cm^−1^ mode provides a more reliable metric of electromagnetic enhancement across different substrates and measurement conditions.

**Figure 7:**
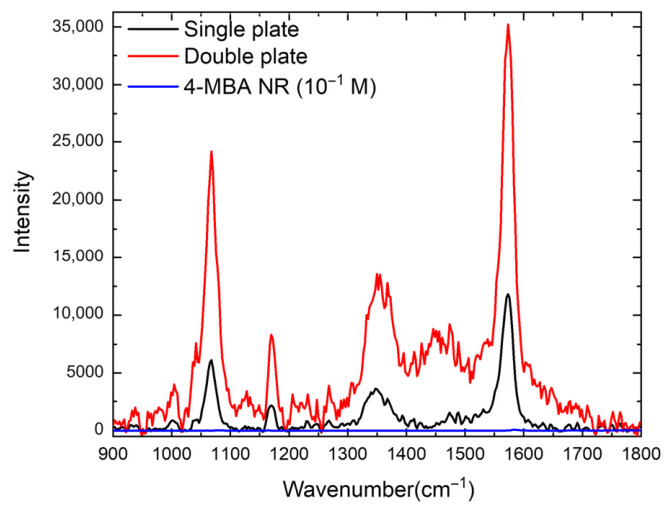
Raman spectrum (blue line) and SERS spectra of 4-MBA on monomers (black line) and dimers (red line).

Both the monolayer and dimer-coated substrates exhibited clear and well-resolved Raman bands corresponding to the aromatic ring vibrations and the C–S stretching modes of 4-MBA (Figure 7), confirming successful adsorption of the analyte. Quantitative analysis of the 1076 cm^−1^ peak yielded average enhancement factors (EFs) of approximately 1.8 × 10^7^ for isolated nanoplates and 6.8 × 10^7^ for the dimer assemblies. These values fall within the range reported for strongly coupled Ag nanoparticle systems, and are consistent with literature trends for nanoparticle dimers [41,42]. Such moderate relative enhancements (3–5×) are typical for rigidly spaced 0.6–0.8 nm gaps and should not be compared to touching or coalesced geometries, where field collapse below ~0.5 nm produces orders-of-magnitude increases.

The nearly fourfold increase in EF correlates with the pronounced strengthening of the near-field in the sub-nanometer junction formed between two stacked NPTs. Although molecules adsorbed from solution may not fully penetrate the narrowest region of the gap, because of the steric constraints imposed by such extreme confinement, even partial occupation of the junction is sufficient to produce a marked increase in Raman intensity because of the |E|^4^ dependence of the SERS process. This behaviour is consistent with previous observations that small increases in the local electric-field amplitude (e.g., 1.4–1.5×) can lead to several-fold increases in the SERS EF, confirming that the measured EF increase is the expected signature of controlled, rigid-gap dimer formation rather than uncontrolled aggregation [43]. Indeed, while partial occupation of the junction can produce a disproportionately large contribution because of the |E|^4^ dependence of SERS, a significant fraction of 4-MBA molecules is also expected to adsorb on the outer surfaces of the nanoplates, where the local fields are weaker but extend over a larger area, thus providing a non-negligible contribution to the overall integrated signal. This interpretation is consistent with the moderate EF increase observed here, which supports a combined response arising from both hotspot-enhanced molecules in or near the gap and more spatially distributed molecules outside the junction.

Accordingly, simulations and analytical plasmon-coupling models predict that for interplate separations of approximately 0.6–0.8 nm, compatible with the extended length of the 1,4-butanedithiol linker, the gap region can sustain a field enhancement larger than that of an isolated NPT by a factor of ~1.4–1.6. When this increased near-field amplitude is combined with the larger optical extinction cross-section of the dimer (acting as a more efficient nanoantenna), the expected |E|^4^ scaling naturally leads to 3–5× stronger SERS enhancement, in good agreement with our measured EF ratio (6.8 × 10^7^/1.8 × 10^7^ ≈ 3.8). Similar behaviour has been reported for nanoparticle dimers in which both antenna effects and gap-mode confinement contribute synergistically to the overall enhancement [42]. To further support the interpretation of the SERS results, FDTD simulations of the near-field intensity distribution were performed for both monomer and dimer configurations (Figure S4a,b). The calculated ∣E∣^2^ maps show that, for the monomer, the field enhancement is mainly localized at the lateral edges of the nanoplate, consistent with an in-plane dipolar plasmon mode. In contrast, the dimer configuration exhibits a much stronger enhancement in both the junction region and in the lateral edges, demonstrating the formation of a plasmonic hotspot associated with coupling between the two stacked nanoplates.

These results demonstrate that the enhanced SERS response of the AgNPT dimers arises not from uncontrolled aggregation or purely dielectric effects, but from the combined action of (i) stronger near-field confinement within the molecular junction and (ii) improved radiative coupling of the plasmonic dimer geometry. The reproducible formation of such well-defined hybrid gap modes provides a robust and controllable platform for quantitative surface-enhanced spectroscopies and for the rational design of hotspot architectures in sensing-oriented nanoplasmonic devices [44].

## 4. Discussion

Our results demonstrate a reproducible and highly controlled strategy for assembling triangular AgNPTs into ordered face-to-face dimers on silica substrates through a sequential APTES anchoring and 1,4-butanedithiol bridging procedure. This two-step functionalization approach enables precise control over NPT orientation, spacing, and structural uniformity, overcoming the limitations typically encountered in random aggregation or solution-phase dimer formation. The AFM height analysis collected over multiple areas (N = 80 objects per sample) provides large-scale statistical validation of the dimer structures, addressing the need for reproducible and quantifiable structural information. At the same time, optical extinction spectroscopy reveals a well-defined interplay between dielectric modulation and plasmonic coupling during each assembly step.

The adsorption of the dithiol linker produces a pronounced redshift of the dipolar LSPR, arising from local refractive-index enhancement at the NPT surface. Subsequent dimerization partially restores the resonance toward shorter wavelengths, consistent with hybridized plasmon modes in strongly coupled nanoparticle dimers. Although the ~0.7 nm gap lies close to the classical–quantum transition and weak nonclassical contributions cannot be excluded, the observed spectral behavior is well reproduced by classical coupling across a rigid molecular spacer, indicating that any quantum effect, if present, plays a secondary role.

Importantly, this method preserves the spectral linewidth and refractive-index sensitivity of individual NPTs while providing an approximately twofold increase in extinction intensity, thereby enhancing the detection signal without compromising spectral resolution.

SERS measurements using 4-MBA confirm that the controlled formation of sub-nanometer interplate gaps leads to a fourfold enhancement in SERS signal intensity from monolayer to bilayer, consistent with the ∣E∣^4^ dependence of the SERS process.

## 5. Conclusions

Overall, this work establishes a robust and scalable platform for engineering NPT assemblies with tunable near-field interactions and enhanced optical response. In particular, the combination of statistical structural validation, controlled plasmonic behavior, and predictable SERS enhancement confirms that the dimers are well-defined and not the result of aggregation-driven artifacts. The approach is compatible with large-area substrates, allows deterministic tuning of interparticle geometry, and yields plasmonic architectures suitable for refractive-index sensing, quantitative SERS, and advanced nanophotonic device design. The methodology also offers a versatile foundation for extending controlled assembly to more complex heterostructures, including multi-plate stacks, mixed-metal systems, and coupled plasmon–exciton platforms.

## Data Availability

The raw data supporting the conclusions of this article will be made available by the authors on request.

## Supplementary Materials

The following supporting information can be downloaded at: https://www.mdpi.com/article/10.3390/nano16080486/s1. Figure S1: SERS spectrum of AgNPTs functionalized with buthadithiol; Figure S2: FDTD Calculated extinction spectra of AgNPTs at increasing refractive index title; Figure S3: the statistical study of the size distribution of 80 nanoparticles for both substrates: monolayer (S3a) and dimer (S3b). Figure S4: FDTD-calculated near-field intensity maps (∣E∣^2^) for the AgNPT monomer (a) and dimer (b) configurations in the x-z plane.

## Abbreviations

The following abbreviations are used in this manuscript:

LSPRs	Localized Surface Plasmon Resonances
AgNPTs	triangular silver nanoplates
AFM	Atomic Force Microscopy
APTES	3-aminopropyltriethoxysilane
4MBA	4-mercaptobenzoic acid
SERS	Surface-enhanced Raman scattering
NIR	near-infrared
