# Supplementary figures and images for: Controlled Plasmonic Coupling in Silver Nanoplate Dimers for Enhanced Plasmonic Sensing
Source: Nanomaterials (Basel). 2026 Apr 19;16(8):486. doi: 10.3390/nano16080486 (PMC13118445; doi:10.3390/nano16080486)

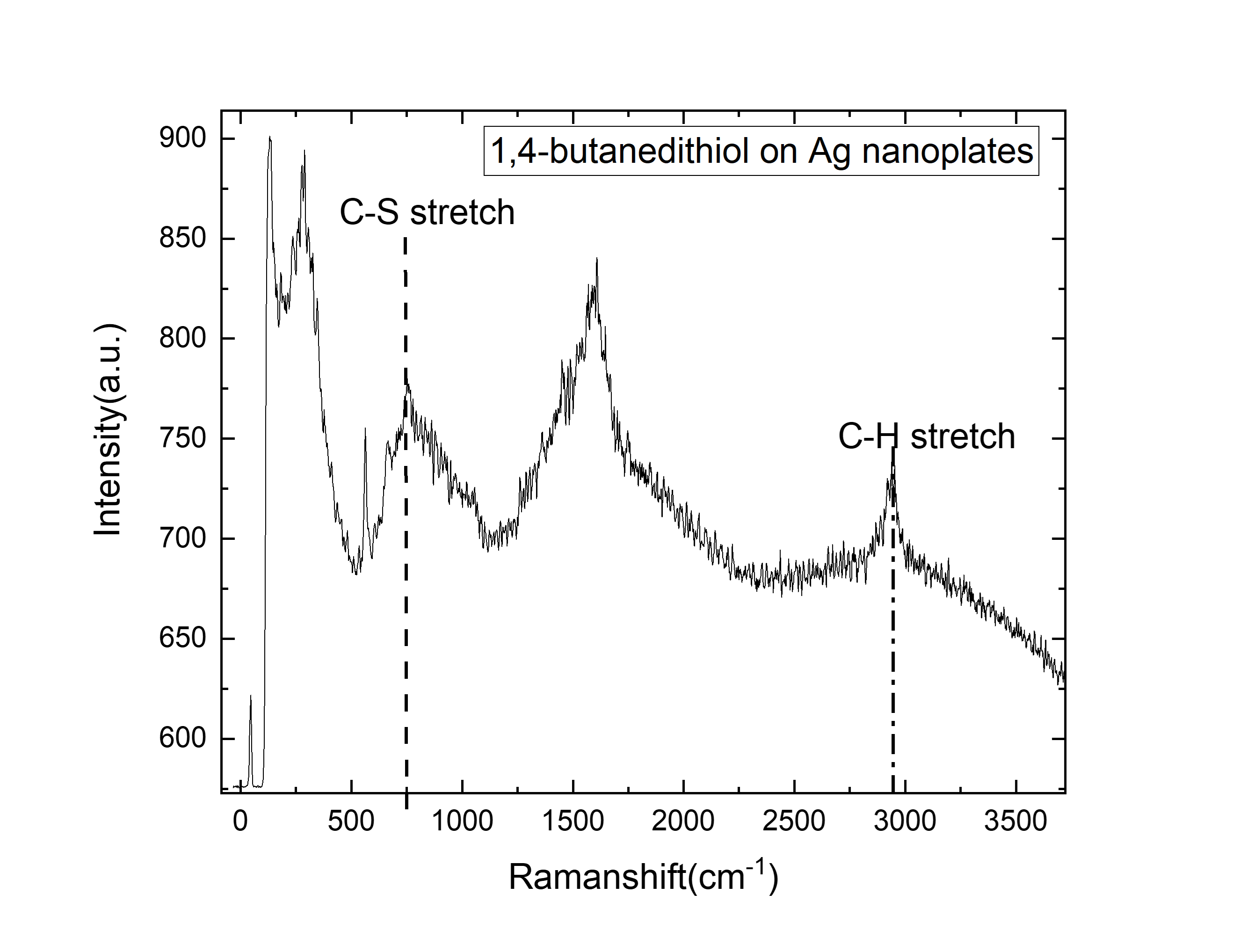

Supplement: Supplementary file 1 [file nanomaterials-16-00486-s001.zip › Figure S1.png]

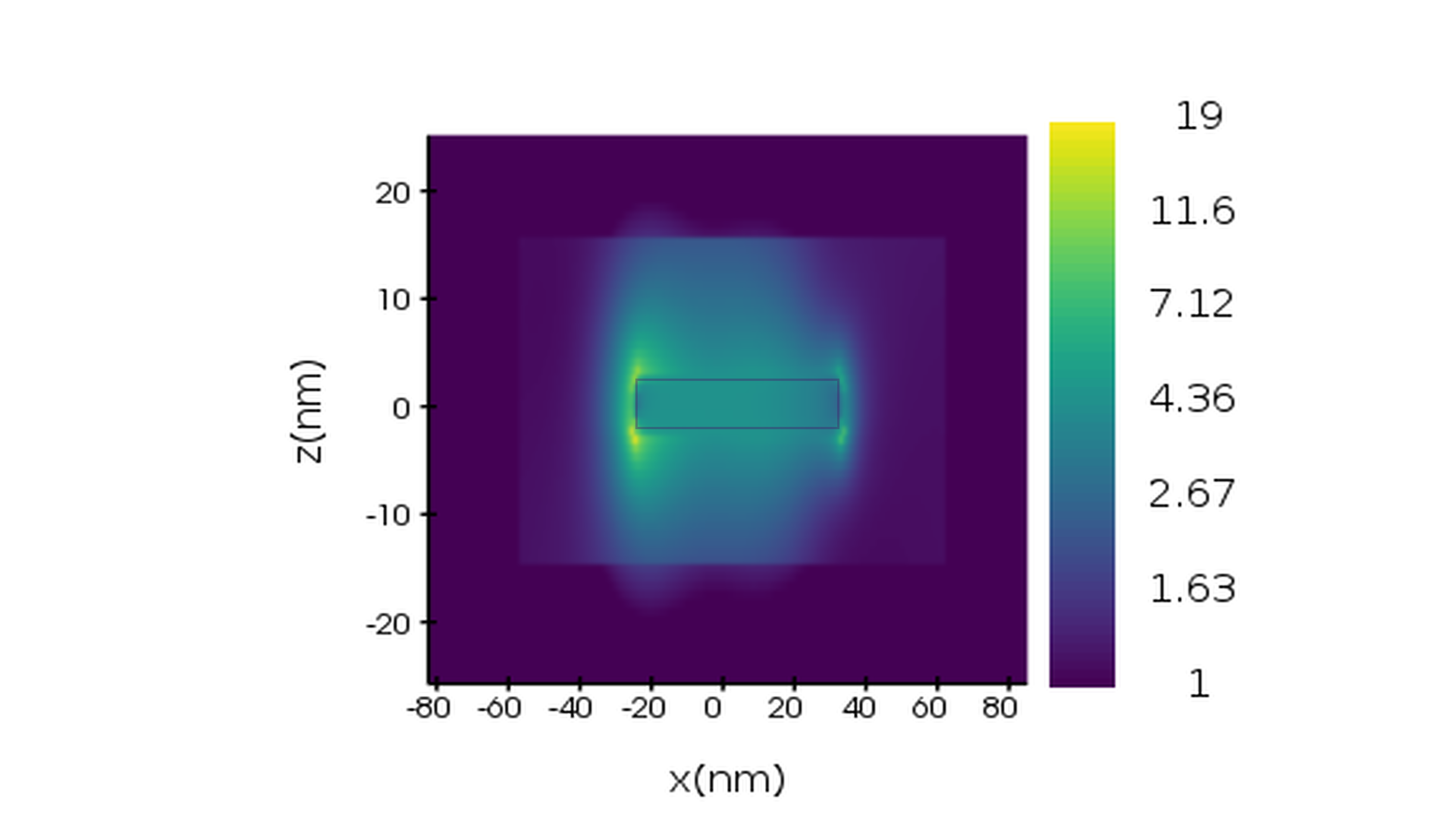

Supplement: Supplementary file 1 [file nanomaterials-16-00486-s001.zip › Figure S4(a).png]

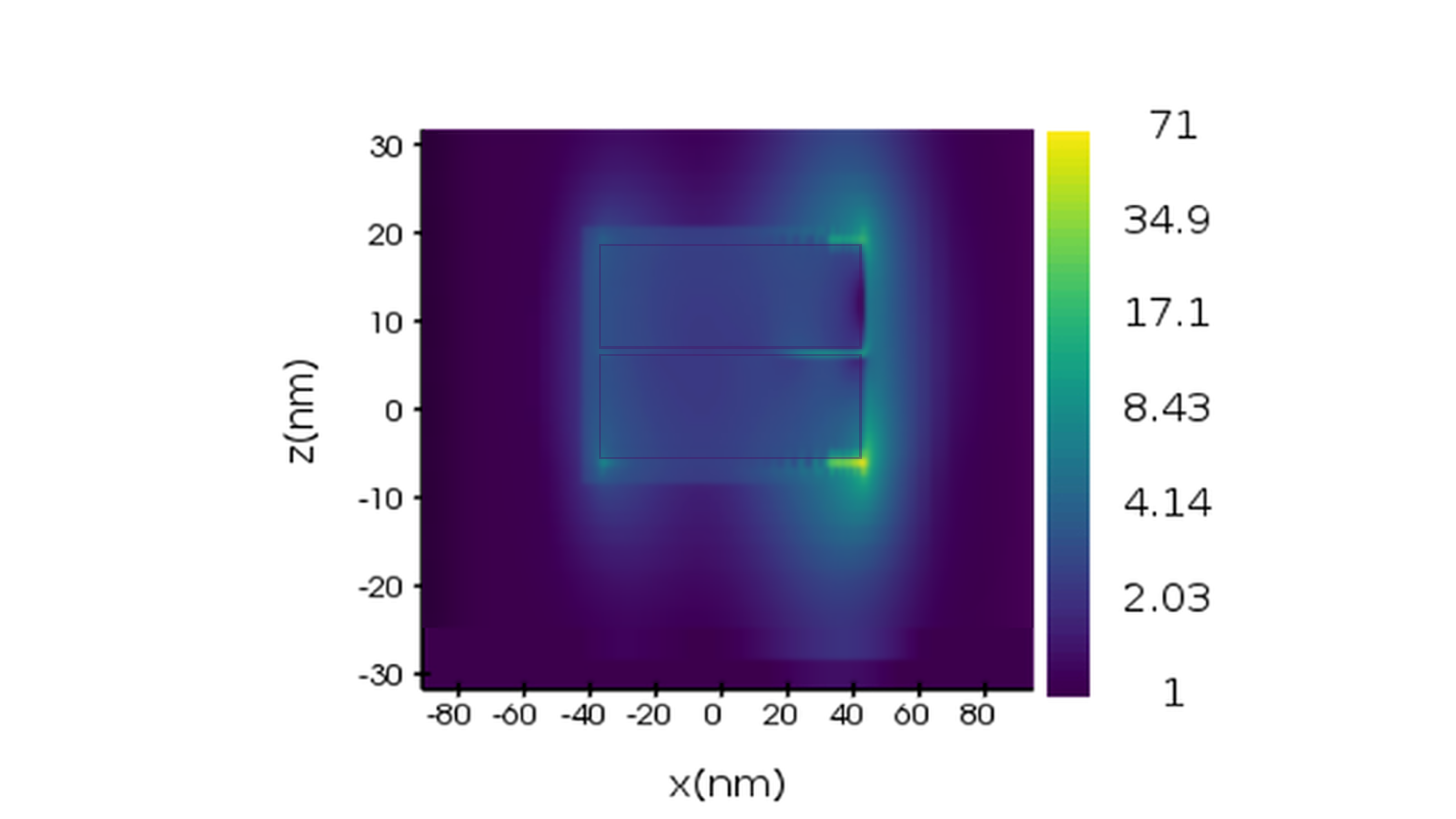

Supplement: Supplementary file 1 [file nanomaterials-16-00486-s001.zip › Figure S4(b).png]
